# Human Endometrial Stromal Stem Cells Differentiate into Megakaryocytes with the Ability to Produce Functional Platelets

**DOI:** 10.1371/journal.pone.0044300

**Published:** 2012-08-31

**Authors:** Jinju Wang, Shuzhen Chen, Cheng Zhang, Samantha Stegeman, Teresa Pfaff-Amesse, Ying Zhang, Wenfeng Zhang, Lawrence Amesse, Yanfang Chen

**Affiliations:** 1 Department of Pharmacology & Toxicology, Boonshoft School of Medicine, Wright State University, Dayton, Ohio, United States of America; 2 Department of Obstetrics & Gynecology, Boonshoft School of Medicine, Wright State University, Dayton, Ohio, United States of America; 3 Department of Obstetrics & Gynecology, Affiliated Hospital of Guangdong Medical College, Zhanjiang, Guangdong, China; Northwestern University, United States of America

## Abstract

Human endometrium is a high dynamic tissue that contains endometrial stromal stem cells (hESSCs). The hESSCs have been differentiated into a number of cell lineages. However, differentiation of hESSCs into megakaryocytes (MKs) has not yet been investigated. The aim of this study was to investigate the feasibility of MK generation from hESSCs and subsequent production of functional platelets (PLTs). In our study, hESSCs were cultured from endometrial stromal cells as confirmed by positive stromal cell specific markers (CD90 and CD29) and negative hematopoietic stem cell markers (CD45 and CD34) expression. Then, hESSCs were differentiated in a medium supplemented with thrombopoietin (TPO) for 18 days. The MK differentiation was analyzed by flow cytometry and confocal microscopy. The differentiation medium was collected for PLT production analysis by flow cytometry, transmission electron microscopy and functional measurements. Our results show: 1) MKs were successfully generated from hESSCs as identified by expression of specific markers (CD41a: 1±0.09% and 39±3.0%; CD42b: 1.2±0.06% and 28±2.0%, control *vs.* differentiation) accompanied with reduction of pluripotent transcription factors (Oct4 and Sox2) expression; 2) The level of PLTs in the differentiation medium was 16±1 number/µl as determined by size (2–4 µm) and CD41a expression (CD41a: 1±0.4% and 90±2.0%, control *vs.* differentiation); 3) Generated PLTs were functional as evidenced by the up-regulation of CD62p expression and fibrinogen binding following thrombin stimulation; 4) Released PLTs showed similar ultra-structure characteristics (alpha granules, vacuoles and dense tubular system) as PLTs from peripheral blood determined by electron microscopic analysis. Data demonstrate the feasibility of generating MKs from hESSCs, and that the generated MKs release functional PLTs. Therefore, hESSCs could be a potential new stem cell source for *in vitro* MK/PLT production.

## Introduction

Platelets (PLTs) play a key role in haemostatic plug formation [1] and arterial thrombosis [2]. PLT transfusion is a mainstay therapy of various PLT-related diseases, such as thrombocytopenia and in cases of menorrhagia that are refractory to medical therapy [3]–[6]. In the United States, the total number of PLT transfusions is over 10 million units per year and is increasing annually. Traditional source of PLTs from blood donors has several limitations, including short storage life of PLT concentrates and risk of pathogenic contamination [7], [8]. Thus, developing a reliable, safe alternative approach to producing large numbers of PLTs is intensely appealing.

Stem cell based generation of megakaryocytes (MKs), the parent cells of circulating PLTs, would provide significant advantages over the current donor-supply approach. So far, embryonic stem cells [9]–[12] and hematopoietic stem cells (HSCs) harvested from bone marrow, peripheral blood and cord blood [13]–[15] have been explored for MK production. However, there are several well-established limitations of these sources, including achieving adequate amounts of HSCs and flawed maturation, as well as political and ethical ramifications.

The endometrium of reproductive-age women is highly dynamic with its remarkable capacity for self-renewal attributed to stromal progenitor or stem cells [16]. Human endometrial stromal stem cells (hESSCs) can be isolated relatively simply from endometrium and maintained in cell culture. The cells can be cultured for more than 15 passages (>20 months) [17]. In addition, successful differentiation of hESSCs into targeted cells has clinical significance because it can provide an autologous cell source that avoids the complications associated with allogeneic transplantation. Indeed, these characteristics render hESSCs a potentially good cell source for regenerative medicine. So far, hESSCs have been reported to be capable of differentiating into mesodermal and ectodermal lineages, including smooth muscle cells [18], adipocytes [17], [18], chondrocytes [18], [19], osteoblasts [18], pancreatic β-cells [20] and neurons [21]. However, to our knowledge, there is no report of differentiating hESSCs into MKs.

In this study, we investigated whether hESSCs could be differentiated into MKs and the generated MKs could release functional PLTs in an *in vitro* culture system.

## Results

### Morphology Observation of hESSC Culture

**Figure 1:**
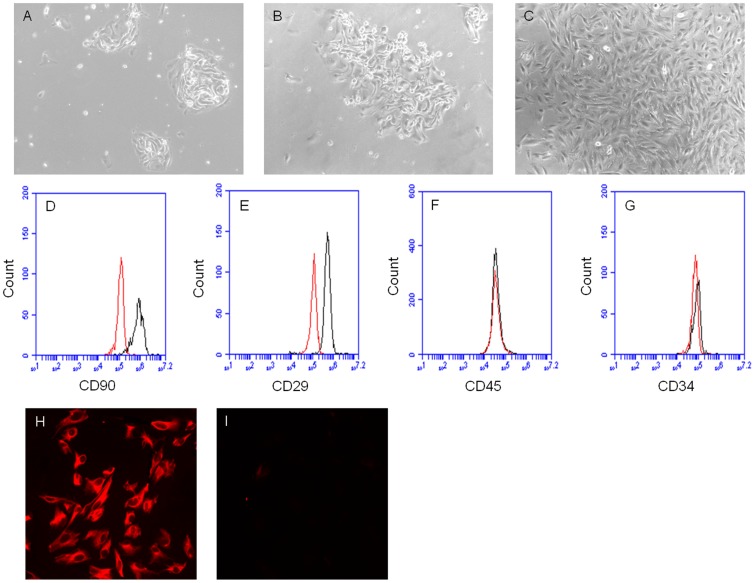
Culture and identification of hESSCs. Representative images (10×) of hESSC culture from endometrial stromal cells at different time points: A, at day 3 (small colonies); B, at day 5 (large colonies); C, at day 10 (90% confluence). Representative flow cytometric plots of hESSCs at 4th passage: D, CD90; E, CD29; F, CD45; G, CD34 (left curves: isotype controls; right curves: antibodies). Representative immunocytochemistry pictures (20×) of hESSCs at 4th passage: H, CD90; I, cytokeratin.

Three days after plating endometrial cells, a significant number of cells attached to the tissue culture plates and grew to small colonies (**Fig1-A**). Then, the small colonies gradually formed large colonies at day 5–6 (**Fig1-B**) and finally achieved 90% confluence at approximately day 8–10 (**Fig1-C**).

### Characterization of hESSCs

The passage 4 and 6 (P_4, 6_) cultured cells were characterized by flow cytometry and immunocytochemistry methods. Flow cytometric results showed that the hESSCs positively expressed stromal cell specific markers CD90 and CD29 (**Fig 1-D, E**) and negatively stained with hematopoietic lineage markers CD45 and CD34 (**Fig 1-F, G**). Immunocytochemistric results revealed that the hESSCs positively stained CD90 (**Fig 1-H**), but negatively stained an epithelial specific marker cytokeratin (**Fig 1-I**).

These results showed that the P_4, 6_ hESSCs was characterized by a homogenous cell population with depletion of hematopoietic and epithelial cells.

### Immunocytochemistry Analysis of MKs Differentiated from hESSCs

**Figure 2:**
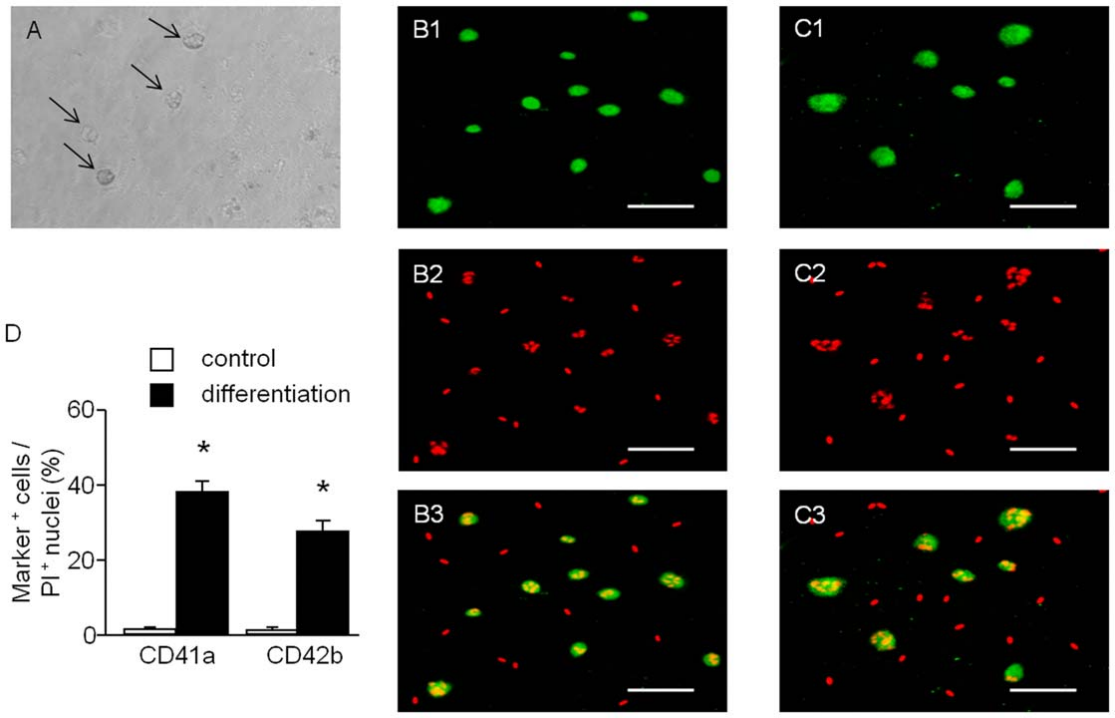
Microscopic analysis of MKs differentiated from hESSCs. MK differentiation can be observed by cell size under light microscope (A: MKs, indicated by black arrows) and determined by immunocytochemistry staining (B1: CD41a; C1: CD42b; B2, C2: PI; B3, C3: merged. scale bar: 75 µm). D, summarized data showing the expression rates are expressed as mean ± SEM, *P<0.01, control versus differentiation, n = 4/group.

The P_4–6_ hESSCs were induced to differentiate into MKs in a serum-free medium in the presence of thrombopoietin (TPO, 50 ng/ml). Generated MKs could be visualized under light microscope (**Fig 2-A**). MK maturation involves the acquisition of characteristic cell surface markers, such as glycoprotein (GP) IIb (CD41a), GPIbα (CD42b). Immunofluorescence studies revealed positive immunoreactivity in generated MKs for the MK specific surface markers CD41a (**Fig 2B_1_–B_3_**) and CD42b (**Fig 2C_1_–C_3_**). The generated MKs showed significantly increased expression rates (CD41a: 1±0.5% and 38±3%; CD42b: 0.9±0.4% and 27±2.5%; control versus differentiation, *P*<0.01, n = 4, **Fig 2-D**) after differentiation.

### Flow Cytometric Analysis of MKs Differentiated from hESSCs

**Figure 3:**
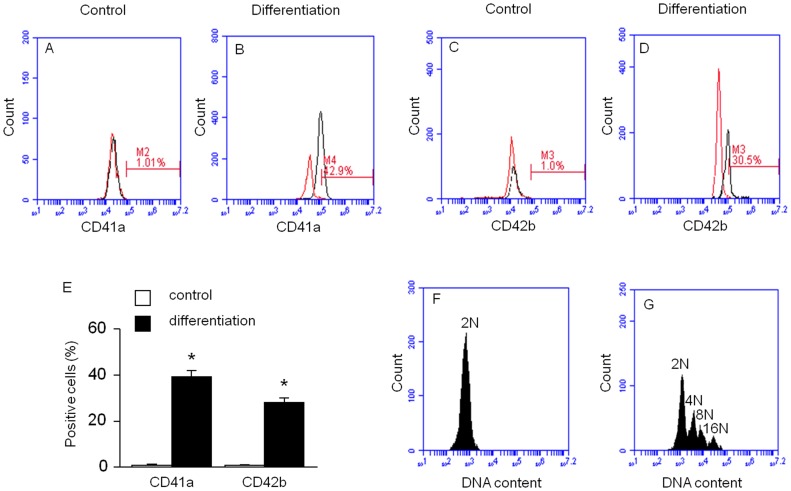
Flow cytometric analysis of MKs differentiated from hESSCs. Flow cytometric analysis of MK differentiation rate was determined by specific cell surface markers (A, B: CD41a; C, D: CD42b; left curves: isotype controls; right curves: antibodies). E, summarized data are expressed as mean ± SEM, *P<0.01, control versus differentiation, n = 4/group. Analysis of DNA polyploidy of the MKs differentiated from hESSCs (F, control; G, MK differentiation).

Differentiated MKs were also detected by flow cytometric analysis of CD41a and CD42b expression. Flow cytometric results revealed that the expression rates of CD41a and CD42b were significantly increased (CD41a: 1±0.09% and 39±3%, **Fig 3-A, B**; CD42b: 1.2±0.06% and 28±2%, **Fig 3-C, D**; control versus differentiation; *P*<0.01, n = 4) after differentiation. These data are summarized in **Fig 3-E**. The calculated number of MKs produced was approximately 5.5×10^4^±500 MKs (CD41a^+^) from 1.8×10^5^±642 hESSCs.

In addition, MK differentiation was identified using DNA ploidy study. We found that cells in the MK differentiation group displayed polyploidy ranged from 2 N to 16 N (**Fig 3-G**), whereas, the cells in control group were diploidy and the DNA ploidy was 2 N (**Fig 3-F**).

### The Levels Oct4 and Sox2 Were Decreased after MK Differentiation

**Figure 4:**
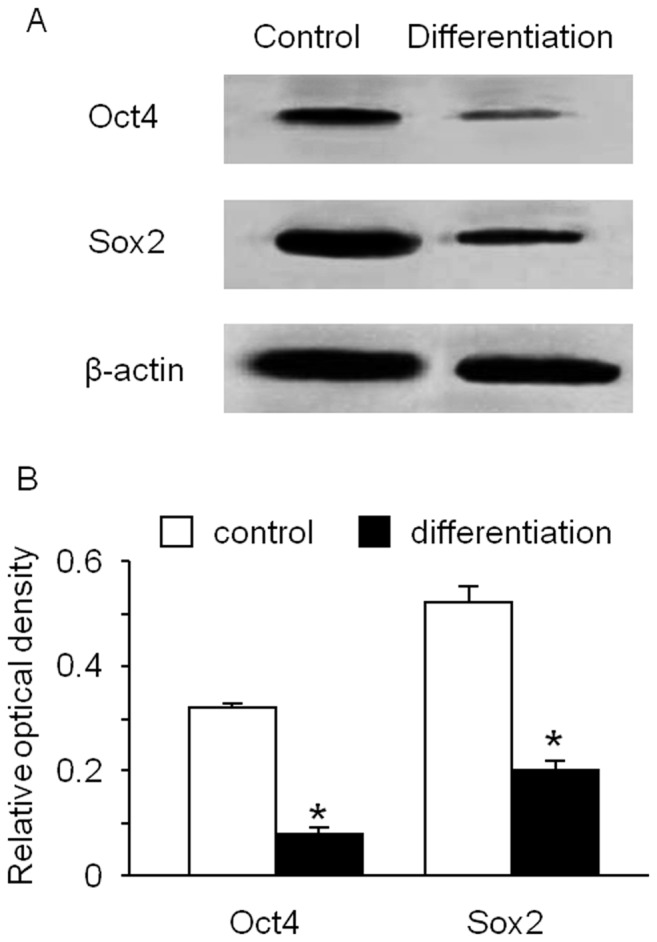
Cellular Oct4 and Sox2 expression after MK differentiation from hESSCs. The expression levels of Oct4 and Sox2 in cells were decreased after induction of MK differentiation (A: representative western blot bands; B: summarized data). Data are expressed as mean ± SEM, *p<0.01, control versus differentiation, n = 4/group.

The expression levels of the pluripotent transcription factors (Oct4 and Sox2) in the cells of MK differentiation and control groups were examined by western blot analysis. The expression levels of both Oct4 and Sox2 were significantly reduced in cells in the differentiation group (Oct4∶0.32±0.01 and 0.08±0.01; Sox2∶0.52±0.03 and 0.20±0.02, control versus differentiation, *P*<0.01, n = 4, **Fig 4-A, B**).

### Differentiated MKs Release PLTs into Culture Medium

**Figure 5:**
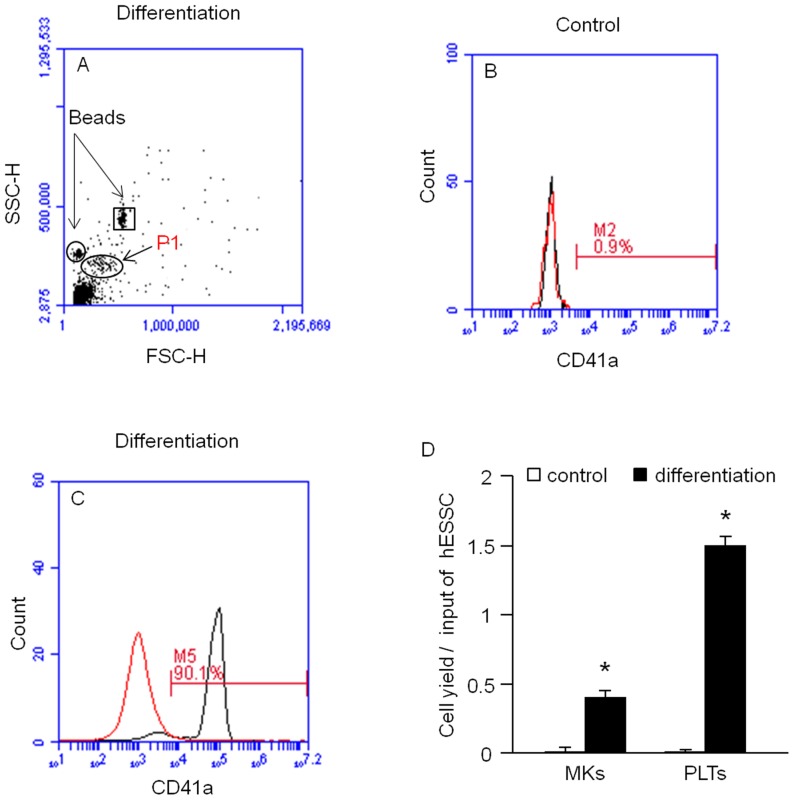
Analysis of PLT production from hESSC-differentiated MKs. Representative flow cytometric plots show the location of PLT population (A, oval indicates the PLT population; circle and square indicate respective locations of 2 µm and 4 µm calibration beads) and the percentage of CD41a expression of the cells gated on P1 (B: control, C: differentiation; left curves: isotype controls; right curves: antibodies). D, summarized data of MK and PLT yield per input of hESSC. Data are expressed as mean ± SEM, *p<0.01, control versus differentiation, n = 4/group.

The differentiation medium was collected for examining the PLTs released from MKs by flow cytometric analysis. The diameter of normal PLTs is approximately 2–3 µm. The gate was fixed in the forward scatter and side scatter histogram by using of 2 µm and 4 µm calibration beads (**Fig 5-A**). Our data indicate a population of cells presented within the gate and had an expression rate of CD41a (1±0.4% and 90±2%, control versus differentiation, *P*<0.01, n = 4, **Fig 5-B, C**). These data demonstrate that PLTs were released from generated MKs. The calculated number of PLTs produced was approximately 3×10^5^±360 PLTs from 1.8×10^5^±642 hESSCs. The level of PLT in the differentitation medium supernatant was 16±1 number/µl.

### Generated PLTs Bind to Fibrinogen and Express CD62p after Thrombin Stimulation

**Figure 6:**
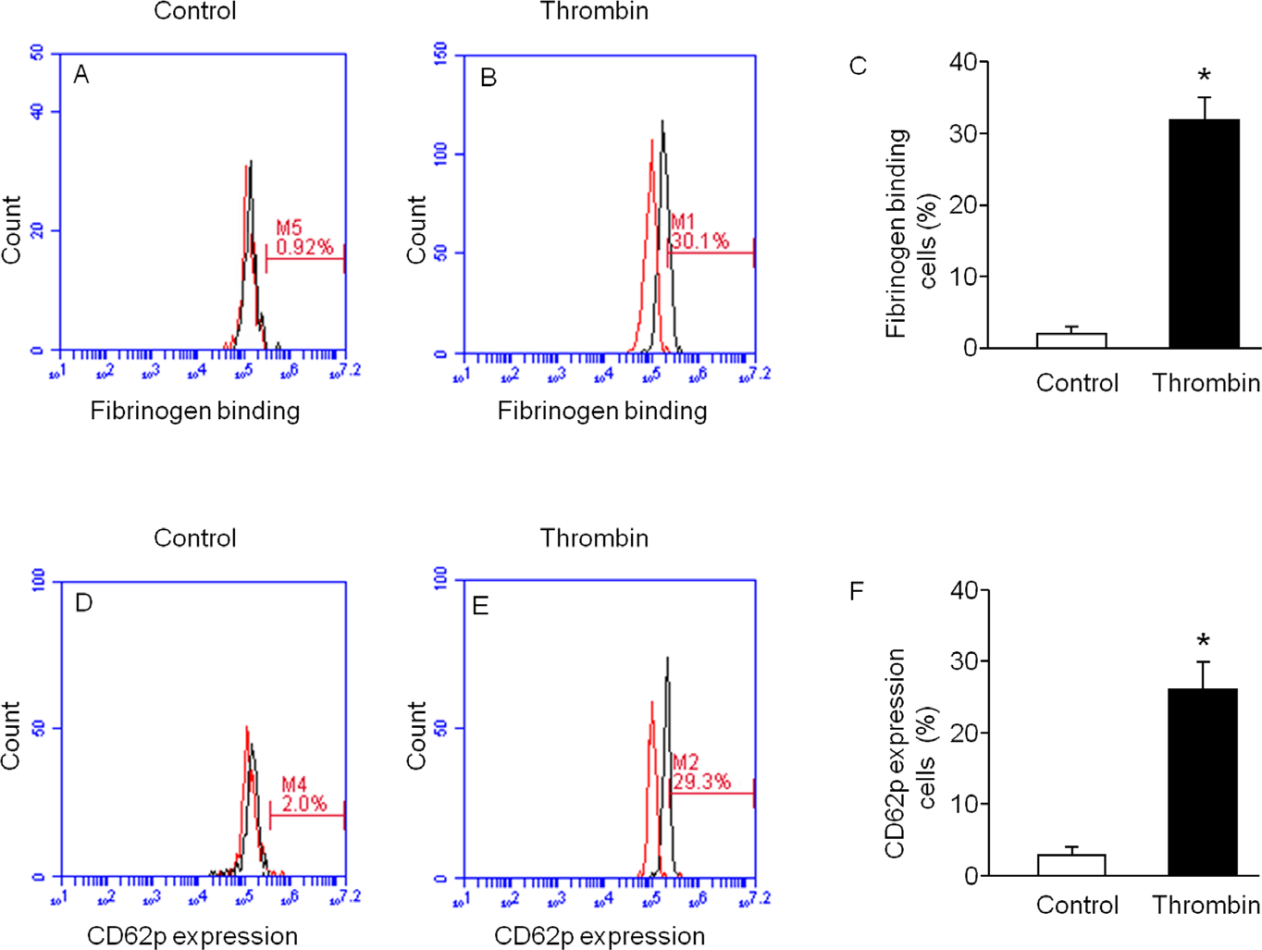
Functional assay of PLTs released from generated MKs. Thrombin stimulation raised fibrinogen binding and CD62p expression in PLTs released from the generated MKs. Representative flow cytometric plots (A, B: fibrinogen binding; D, E: CD62p. left curves, isotype controls; right curves, antibodies). Summarized data show fibrinogen binding (C) and CD62p expression (F) in PLTs after thrombin stimulation. Data are expressed as mean ± SEM, *P<0.01, control versus stimulation, n = 4/group.

The function of the released PLTs was determined by thrombin (5 U/ml, 10 min) stimulation in the presence of 488-labeled fibrinogen. We found the fibrinogen binding rate was increased (1±0.4% and 32±3%, control versus thrombin, *P*<0.01, n = 4) after thrombin stimulation (**Fig 6-A, B**). These data are summarized in **Fig 6-C**. The function of the released PLTs was also detected by the expression of P-selectin (CD62p) with thrombin stimulation. And the results showed the CD62p expression rate was up-regulated (2.5±1% and 26±4%, control versus thrombin, *P*<0.01, n = 4) after thrombin stimulation (**Fig 6-D, E**). These data are summarized in **Fig 6-F**. Our data from thrombin stimulation studies revealed that PLTs released from hESSC-differentiated MKs were functional.

### PLTs Released from Differentiated MKs Share Similar Ultra-structural Characteristics with Peripheral Blood Derived PLTs

**Figure 7:**
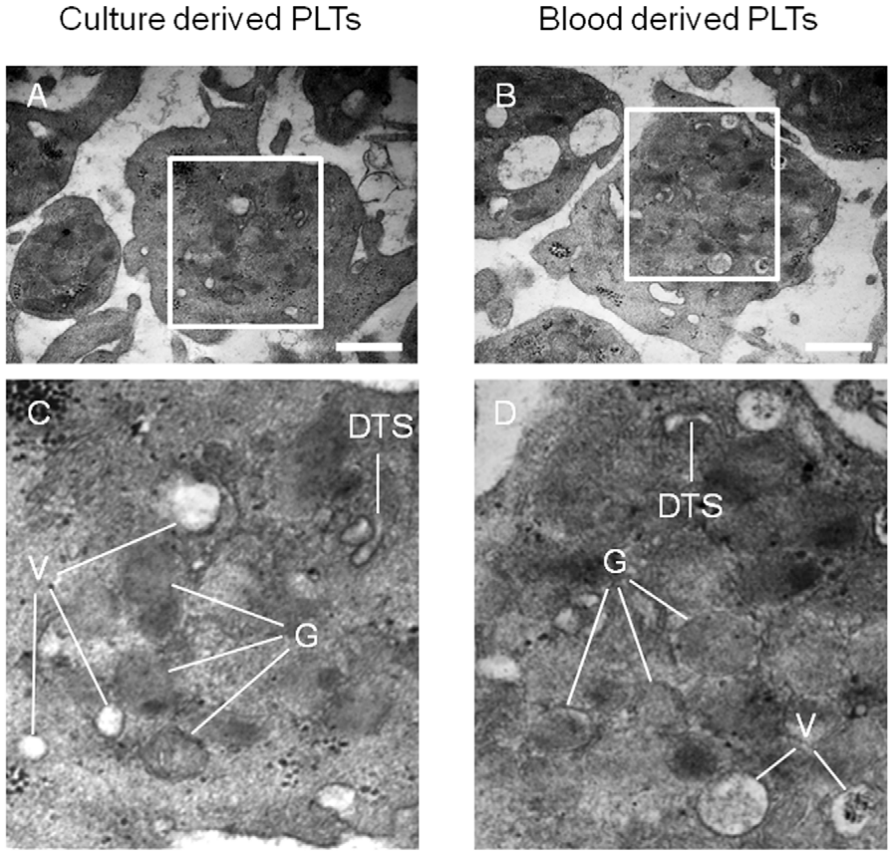
Ultra-structural analysis of released PLTs. Electron micrographs show that generated PLTs have typical ultra-structural morphology and cellular organelles (alpha granules, vacuoles and dense tubular system) as PLTs from peripheral blood. A: generated PLT; B: blood PLT; C: magnified view from A; D: magnified view from B; G: alpha granule; V: vacuole; DTS: dense tubular system. Scale bars: 0.5 µm in A and B.

The morphology and ultra-structural composition of PLTs released from generated MKs was examined by transmission electron microscopy. A representative image of a generated PLT, shown in **Fig 7-A**, displayed a discoid shape and was similar to the discoid shape characteristic of peripheral blood PLTs (**Fig 7-B**). Magnified views show the presence of alpha granules, vacuoles and dense tubular system in PLTs released from differentiated MKs (**Fig 7-C**). These intracellular structures are similar to PLTs harvested from human peripheral blood (**Fig 7-D**).

## Discussion

In this study, we developed a protocol to effectively induce the differentiation of hESSCs into MKs. Generated MKs were able to release functional PLTs with size and ultra-structure characteristics similar to normal PLTs obtained from peripheral blood. Combined methodologies such as immunocytochemistry, flow cytometry and transmission electron microscopy were applied to characterize the generated MKs or PLTs.

Human endometrium is characterized by its cyclic self-regeneration during the course of menstrual cycle in reproductive-age females. The upper functionalis layer (0.5–1 mm to 5–7 mm thickness), which sheds at menstruation is thought to regenerate from stem cells in the basalis layer of endometrium. The existence of hESSCs has been investigated in several previous studies. Initially, Chan and coworkers [22], [23] demonstrated the presence of clonogenic stromal stem cells with high proliferative potential in active cycling and inactive endometrium. And their high proliferation rate was preserved despite their long duration in culture (more than 20 months) [17]. These innate characteristics suggest that hESSCs may have advantages over other adult stem/progenitor cells and may represent a new resource for cell-based therapies and regenerative medicine. Recent studies have been shown that hESSCs are able to differentiate into multiple cell lineages, including adipocytes, chondrocytes, osteoblasts, neurons and pancreatic β-cells [17]–[21]. However, differentiation of hESSCs into MKs has not been reported yet.

This study is the first time to describe a culture system in which MKs was differentiated from hESSCs in a serum-free medium supplemented with TPO. In this study, P_4–6_ hESSCs were used for the differentiation experiment. These hESSCs was a homogenous cell population as evidenced by highly positive expression of stromal cell markers (CD29 and CD90), negative expression of hematopoietic stem cell markers (CD34 and CD45) and an epithelial marker cytokeratin. After 18 days differentiation, the hESSCs-derived MKs displayed polyploidy with the DNA ploidy ranged from 2 N to 16 N, accompanying with a significantly increased expression profile of characteristic MK specific plasma membrane glycoprotein markers (e.g., CD41a, CD42b). In addition, since Oct4 and Sox2 are pluripotent human stem cell markers detected in endometrium and other adult stem cells [24]–[26], we also verified the differentiation of hESSCs by measuring the expression profile of Oct4 and Sox2. Our data indicated that the expression levels of Oct4 and Sox2 were both significantly reduced after differentiation, indicating their phenotype paralleled the molecular changes associated with differentiation.

In order to further explore whether the differentiated MKs were able to release PLTs, MK differentiation medium were collected to detect PLT production by analysis of specific marker CD41a and size (2–4 µm). Our data showed that the level of released PLTs in the culture medium was around 16 number/µl. Moreover, we functionally evaluated the produced PLTs with two thrombin stimulation studies. One is thrombin stimulation induced activation of fibrinogen receptor (αII_b_III_a_), which is an integrin complex found on PLTs and aids in PLT activation by conformational change. The result showed the fibrinogen binding rate significantly increased from 1±0.4% to 32±3% with the stimulation. The other is thrombin stimulation induced surface expression of antigen P-selectin (CD62p). The result showed the percentage of specific CD62P^+^ PLTs significantly increased from 2.5±1% to 26±4% with the stimulation. Both stimulation studies demonstrate that the released PLTs from generated MKs are functional in our culture system. In addition, we applied transmission electron microscopy (TEM) to examine the ultra-structures of generated PLTs. The TEM examination results revealed that the produced PLTs displayed a discoid shape and contained a normal distribution of organelles such as alpha granules, vacuoles and dense tubular system, which are indistinguishable from the PLTs obtained from peripheral blood. These data are consistent with previous studies on *in vitro* generated MKs and PLTs from other tissue of origin sources [9], [12], [27]–[29]. Despite further studies are required to determine the optimal protocols for MK differentiation from hESSCs, as well as for PLT release from MKs, this study for the first time reports that MKs can be feasibly generated from hESSCs and that the generated MKs can release functional PLTs. PLTs generated in such an approach could be applied to autologous PLT transfusion. Our future work is to examine whether hESSCs induced inducible pluripotent stem cells (iPSCs) could further increase the efficiency of MK differentiation and PLT production.

### Conclusion

In conclusion, the results of our study demonstrate that hESSCs can differentiate into MKs which subsequently release functional PLTs in an *in vitro* culture system. The hESSCs have the potential to serve as a new source of PLT production for clinical application.

## Materials and Methods

### Ethics Statement

For this study, ethics approval was obtained from the Institutional Review Boards and Research Committees at the Miami Valley Hospital and Wright State University. Written informed consent was obtained from each patient and the patient confidentiality was protected according to HIPPA guidelines.

### Human Endometrial Tissue Samples

Four premenopausal women (30–45 years old) who were undergoing hysterectomy for benign disease indications, leiomyoma, were selected. The full thickness endometrium attached to myometrium sample was collected and put into PBS containing 10% FBS and 2% antibiotic-antimycotic solution (final concentration: 200 µg/ml penicillin G sodium and 200 µg/ml streptomycin sulfate) (Invitrogen, Carlsbad, CA). The sample was processed within 30–60 min.

### Isolation and Culture of hESSCs

As soon as the sample was delivered to the laboratory, the endometrial tissue was mechanically and enzymatically digested into a single-cell suspension as previously reported with slight modifications [17], [22]. Briefly, the endometrium layer was dissected, minced into 1–2 mm^3^ pieces, and digested in enzyme digestion buffer, PBS with 25 µM HEPEs (Invitrogen, Carlsbad, CA), 1 mg/ml collagenase (Roche Applied Science, Indianapolis, IN), 0.1 mg/ml DNAse1(Sigma, Fairfax, VA) and 1% antibiotic-antimycotic solution, in a humidified 5% CO_2_/95% air atmosphere at 37°C for 45 min with a brief shaking once every 5 min. The cell suspension was filtered through a 70 µm wire sieve to remove mucus and undigested tissue. The filtrates were centrifuged at 400 × g for 10 min to collect cells. Cell pellets were resuspended with hESSCs culture medium, DMEM/F12 (Invitrogen, Carlsbad, CA) supplemented with 10% FBS and 1% antibiotic-antimycotic solution. The cells were seeded in a 6-well tissue culture plate in a humidified 5% CO2/95% air incubator. The medium was changed twice a week during expansion of the cells. When the cells were 90% confluent, they were detached using 0.025% trypsin/0.01% EDTA and passaged at a ratio of 1∶3.

### Identification of hESSCs

Passage 4–6 endometrial stromal cells were used for identifying hESSCs by immunocytochemistry and flow cytometry analyses.

For immunostaining, the cultured cells on coverslips were fixed with 2% PFA solution and stained with monoclonal antibodies mouse anti-human CD90 (Thy-1, 1∶25) (BD Biosciences, San Jose, CA) and mouse anti-human cytokeratin (1∶50, Dako, Carpinteria, CA) followed by a Cy3-conjugated donkey anti-mouse IgG second antibody (1∶250, Jackson lmmunoResearch Lab, West Grove, PA). Then the cells were mounted with fluorescence mounting medium and imaged under a fluorescence microscope (Leica TCS SP2, Leica, Germany).

For flow cytometry, the cultured cells were trypsinized and centrifuged. Cell pellets were resuspended and incubated with PE- or FITC-conjugated antibodies in a final volume of 100 µl for 30 min at 4°C in the dark, individually. Isotype matched (IgG) non-specific antibodies served as negative controls. Antibodies (PE-conjugated anti-human CD29, PE-conjugated anti-human CD90, FITC-conjugated anti-human CD45, FITC-conjugated anti-human CD34, FITC-conjugated mouse IgG1 isotype control, PE-conjugated mouse IgG1 isotype control) were purchased from eBioscience (San Diego, CA). The concentrations of antibodies were applied according to manufacture instructions. A total of at least 10 000 events were collected and analyzed using Accuri C6 flow cytometer and CFlow Plus Analysis software (Ann Arbor, MI).

### Differentiation of MKs from hESSCs

The P_4–6_ of hESSCs were plated as 1.8×10^5^ cells/well on 6-well tissue culture plates or coverslipes. The cells were cultured in Iscove’s modified dulbecco’s medium (IMDM, Invitrogen, Carlsbad, CA) supplemented with 50 ng/ml TPO (BD Biosciences, San Jose, CA) in differentiation group or without TPO in control group, 0.5% BSA (Sigma, Fairfax, VA), 200 µg/ml iron saturated transferring (Invitrogen, Carlsbad, CA), 10 µg/ml insulin (Sigma, Fairfax, VA), 2 mM L-glutamine (Invitrogen, Carlsbad, CA), 4 µg/ml LDL cholesterol (Sigma, Fairfax, VA), 50 µM 2-β-mercaptoethanol (Sigma, Fairfax, VA), 20 µM each nucleotide (Sigma, Fairfax, VA), 20 µM dNTP (Invitrogen, Carlsbad, CA), and 1% antibiotic-antimyotic solution for 18 days. Half of the culture medium was replaced in the first 9 days. The culture medium (2 ml) from day 10–18 was collected for PLT isolation. Cells on culture plates were harvested for flow cytometry or western blotting analysis. Cells on coverslips were used for MK immunocytochemistry assay.

### Characterization of MK Differentiation by Immunocytochemistry Assay

MK differentiation was determined by analysis of surface specific markers (CD41a and CD42b). For immunostaining, the cultured cells on coverslips were fixed with 2% PFA solution for 30 min at RT, and permeabilized with 0.3% triton X-100 for another 30 min. Then slides were incubated overnight with pre-diluted primary antibody CD41a (glycoprotein αIIb) or CD42b (glycoprotein αIb, both from eBioscience, San Diego, CA), and FITC-conjugated anti-mouse IgG secondary antibody (1∶100, Jackson lmmunoResearch Lab, West Grove, PA) for 2 hrs. Cell nuclei were stained with propidium iodide (PI) solution containing RNAse (PI/RNAse final concentration: 20 µg/ml, Sigma, Fairfax, VA) for 15 min in the dark. Between each step, slides were washed with PBS/0.1% BSA. Cells were examined by confocal fluorescence microscopy (Leica TCS SP2, Leica, Germany). To assess the differentiation rate, the numbers of CD41a or CD42b positive staining cells in 5 individual fields were accounted and averaged.

### Characterization of MK Differentiation by Flow Cytometry

Surface specific markers (CD41a and CD42b) were also used for flow cytometric analysis of MK differentiation. The cells were detached, centrifuged and fixed with 2% PFA. Then, the cells were permeabilized with blocking buffer containing 0.3% triton X-100, incubated with FITC-conjugated antibodies in a final volume of 100 µl for 30 min at 4°C in the dark, respectively. Antibodies (FITC-conjugated anti-CD41a, FITC-conjugated anti-CD42b, FITC-conjugated mouse IgG1 isotype controls) were purchased from BD Biosciences (San Jose, CA). The concentrations of antibodies were applied according to manufacture instructions. Cell nuclei were stained with PI solution containing RNAse (PI/RNAse final concentration: 20 µg/ml) for 30 min in the dark. A total of at least 150 000 events were collected and analyzed using an Accuri C6 flow cytometer and CFlow Plus Analysis software (Ann Arbor, MI).

### Characterization of MK Differentiation by Western Blot Analysis of Oct4 and Sox2 Expression

After hESSCs were cultured in MK differentiation or control medium for 18 days, cells were harvested with lysis buffer. Cell lysates were then centrifuged at 10 000 × g for 5 min. Protein concentrations in the supernatants were determined by the Bradford method using Bio Rad reagent (Bio Rad Laboratories) and the same amount of protein (30 µg) from each was separated by 10% Tris-Glycine SDS-Page gel (Bio Rad Laboratories) and transferred to PVDF membrane. After blocking with 5% non-fat milk for 1 hr at RT, membranes were incubated for primary monoclonal antibodies rabbit anti-Oct4 (1∶500, Abcam Inc, Cambridge, MA) and mouse anti-Sox2 (1∶2000, R&D systems, Minneapolis, MN) overnight at 4°C, respectively. After 3 times wash, secondary antibodies horse raddish peroxidase (HRP) conjugated goat anti rabbit (1∶4000) and HRP-conjugated donkey anti mouse (1∶4000, both were from Jackson lmmunoResearch Lab, West Grove, PA) were probed, individually. Then the signal was read with a luminescent analyzer (LAS-3000, Fujifilm, Japan) and the band intensities were analyzed by ImageGauge (Fujifilm, Japan).

### Flow Cytometric Analysis of PLT Production in Culture Medium

To determine the number of PLT released, we collected the culture medium (2ml) of the same well of a 6-well culture plate every day from day 10–18 of the differentiation group or control group. The collected culture medium was centrifuged at 150 × g for 20 min to eliminate any large cells each time. The supernatant was fixed with 2% PFA, centrifuged at 1700 × g for 30 min to sediment a PLT pellet. The pellet was resuspended with PBS/1% FBS, and stored at 4°C for further analysis. For analysis, the fixed and stored PLT pellets from the same well were pooled together and then divided into two groups. One group was resuspended and incubated with FITC-conjugated anti-CD41a antibody in a final volume of 100 µl for 30 min at 4°C in the dark. The other group was incubated with Isotype matched (IgG) non-specific antibody in the same amount of volume (100 µl) and served as negative control. 2 µm and 4 µm flow cytometry calibration bead solution was added into each tube. The concentrations of calibration beads and antibodies were applied according to manufacture instructions. A total of at least 10 000 events within the gate were collected and analyzed using the Accuri C6 flow cytometer. The concentration of PLT (CD41a^+^) in the culture medium supernatant of each well was calculated as: PLT numbers/µl of culture medium  =  [CD41a^+^ numbers × 100 µl/actual run volume (µl)]/total culture medium volume (2 × 9 × 10^3^ µl) × groups (2).

### Functional Analysis of Released PLTs

The functional capabilities of generated PLTs were analyzed for both fibrinogen binding and surface antigen CD62P expression in the presence or absence of thrombin stimulation. To prepare cell samples, PLTs were harvested from the culture medium, centrifuged at 150 × g for 20 min to remove large cells, resuspended and then centrifuged at 1700 × g for 30 min to sediment a PLT pellet as above described. The PLT pellets were resuspended in modified Tyrode-HEPEs buffer (138 mM NaCl, 0.36 mM NaH_2_PO4, 2.9 mM KCl, 12 mM NaHCO_3_, 10 mM HEPEs, 5 mM glucose, 1 mM MgCl_2_, and 1 mM CaCl_2_, pH 7.4).

To determine fibrinogen binding, generated PLTs were stimulated with agonist thrombin (5 U/ml, Sigma, Fairfax, VA) for 10 min at 37°C in the presence of Alexa Fluor 488-labeled human fibrinogen (100 µg/ml, Invitrogen, Carlsbad, CA) or the isotype control mouse IgG. For controls, generated PLTs without thrombin stimulation were also stained with Alexa Fluor 488-labeled fibrinogen or its corresponding isotype control IgG.

To determine the surface expression of P-selectin (CD62p, eBioscience, San Diego, CA), after thrombin stimulation, the generated PLTs were fixed with 2% PFA and incubated with FITC-conjugated anti-CD62p or the isotype control mouse IgG. For controls, generated PLTs without thrombin stimulation were also stained with FITC-conjugated anti-CD62p or its corresponding isotype control IgG. Final volume per tube was brought to 100 µl. A total of at least 100 000 events were collected each and all samples were analyzed by Accuri C6 flow cytometer. All antibody application concentrations followed manufacture instructions.

### Ultra-structure Analysis of Generated PLTs

PLT specimens for ultra-structural study by TEM were prepared as described previously with slight modifications [9], [30]. Briefly, generated PLTs in culture medium supernatant were collected as above described. Peripheral blood samples were collected from the health donors using standard phlebotomy techniques into a collection tube containing acid-citrated-dextrose (ACD) solution. The blood samples were centrifuged at 150 × g for 20 min to obtain PLT-rich plasma (PRP). Blood PLTs were fixed in 2% glutaraldehyde in 0.1 M phosphate buffer for 1 hr at 4°C. The samples were then washed, post-fixed with 1% osmium tetroxide (Electron Microscopy Science, Hatfield, PA) in 0.1 M phosphate buffer for 2 hrs at 4°C, dehydrated with a graded ethanol series and propylene oxide (Sigma, Fairfax, VA), and embedded in spurr resin 812 (Sigma, Fairfax, VA) and baked. Ultrathin sections (60–80 nm) were prepared with MT700, mounted on 300-mesh copper grids, stained with 2% uranyl acetate (Fisher Scientific, Pittsburgh, PA) for 5 min, rinsed with double distilled water for 5 times, and allowed to dry. The specimen were then stained with lead citrate (Fisher Scientific, Pittsburgh, PA) for 10 min, rinsed with double distilled water another 5 times and allowed to air dry. Finally, all specimens were examined with an EM 208 (Philips) transmission electron microscope at an accelerating voltage of 70 KV.

### Statistic Analysis

Results were described as mean ± SEM. The p value <0.05 was considered to be statistically significant in the paired t-test.
